# *In Vitro, In Silico* and *In Vivo* Studies of Ursolic Acid as an Anti-Filarial Agent

**DOI:** 10.1371/journal.pone.0111244

**Published:** 2014-11-06

**Authors:** Komal Kalani, Vikas Kushwaha, Pooja Sharma, Richa Verma, Mukesh Srivastava, Feroz Khan, P. K. Murthy, Santosh Kumar Srivastava

**Affiliations:** 1 Medicinal Chemistry Department, CSIR-Central Institute of Medicinal and Aromatic Plants, Lucknow, 226015 (U.P.) India; 2 Division of Parasitology, CSIR-Central Drug Research Institute, Lucknow, 226001, UP, India; 3 Metabolic & Structural Biology Department, CSIR-Central Institute of Medicinal and Aromatic Plants, Lucknow, 226015 (U.P.) India; 4 Clinical and Experimental Medicine, Biometry section, CSIR-Central Drug Research Institute, Lucknow, 226001, UP, India; 5 Academy of Scientific and Innovative Research (AcSIR), Anusandhan Bhawan, New Delhi, 110 001, India; University of Illinois, United States of America

## Abstract

As part of our drug discovery program for anti-filarial agents from Indian medicinal plants, leaves of *Eucalyptus tereticornis* were chemically investigated, which resulted in the isolation and characterization of an anti-filarial agent, ursolic acid (UA) as a major constituent. Antifilarial activity of UA against the human lymphatic filarial parasite *Brugia malayi* using *in vitro* and *in vivo* assays, and *in silico* docking search on glutathione-s-transferase (GST) parasitic enzyme were carried out. The UA was lethal to microfilariae (mf; LC_100_: 50; IC_50_: 8.84 µM) and female adult worms (LC_100_: 100; IC50: 35.36 µM) as observed by motility assay; it exerted 86% inhibition in MTT reduction potential of the adult parasites. The selectivity index (SI) of UA for the parasites was found safe. This was supported by the molecular docking studies, which showed adequate docking (LibDock) scores for UA (−8.6) with respect to the standard antifilarial drugs, ivermectin (IVM −8.4) and diethylcarbamazine (DEC-C −4.6) on glutathione-s-transferase enzyme. Further, *in silico* pharmacokinetic and drug-likeness studies showed that UA possesses drug-like properties. Furthermore, UA was evaluated *in vivo* in *B. malayi*-*M. coucha* model (natural infection), which showed 54% macrofilaricidal activity, 56% female worm sterility and almost unchanged microfilaraemia maintained throughout observation period with no adverse effect on the host. Thus, in conclusion *in vitro, in silico* and *in vivo* results indicate that UA is a promising, inexpensive, widely available natural lead, which can be designed and developed into a macrofilaricidal drug. To the best of our knowledge this is the first ever report on the anti-filarial potential of UA from *E. tereticornis*, which is in full agreement with the Thomson Reuter's ‘Metadrug’ tool screening predictions.

## Introduction

Among the six neglected tropical diseases, lymphatic filariasis (LF) is one of the major health problems in 73 tropical and subtropical countries in Africa, Asia, South and Central America and the Pacific Islands. According to the World Health Organization (WHO) global report, over 120 million people are currently infected with LF [1], [2] of which about 40 million people are suffering with chronic disease manifestations: Elephantiasis and hydrocele [3], which cause permanent, long-term disability and economic loss to the nations [3], [4]. The LF is caused by the nematode parasites *Brugia malayi, B. timori* and *Wuchereria bancrofti* and according to a recent report about 1 billion people (18% of the world's population) are at risk of infection (www.globalnetwork.org). Although, the World Health Organization launched a global filariasis elimination programme [5], [6] using diethylcarbamazine (DEC) or ivermectin (IVM), but due to serious technical difficulties the programme is facing problem in the eradication of this endemic disease [4], [7]–[8]. Since, DEC and IVM both are microfilaricides with poor or no activity on adult parasites [9], the peripheral blood microfilaremia reappears in patients after a certain period of withdrawal of the drug. This depressing perspective demands, an urgent need for new molecular structures associated with macrofilaricidal activity/or sterilizing the adult worms is therefore needed [8]–[10] as adult parasites not only produce millions of microfilariae (mf) that are picked up by the mosquito vector and transmitted, but are also responsible for the debilitating pathological lesions. Therefore, macrofilaricidal agents are the need of hour, which not only adversely affect the target but should have also very low or no side effect [11].

As a part of our drug discovery program, we recently reported a pentacyclic triterpenoid, glycyrrhetinic acid [9] as a novel class of anti-filarial agent. This prompted us to investigate anti-filarial activity in other pentacyclic triterpenoids, widely available in Indian medicinal plants. For this purpose, in the present study leaves of *Eucalyptus tereticornis* were chemically and biologically investigated in details, which afforded an anti-filarial agent, Ursolic acid (UA, a pentacyclic triterpenoid) as a major constituent. The *in-vitro* activity of UA against the mf and adult worms, *in-silico* docking studies on glutathione-s-transferase (GST) parasitic enzyme and *in vivo* activity against *B. malayi in Meriones unguiculatus* model have been discussed here in detail.

## Materials and Methods

### General experimental procedure

The ^1^H and ^13^C NMR spectra were recorded on a Bruker 300 MHz spectrometer in deuterated pyridine. ESI-MS was carried out on a LCMS-2010 V (Shimadzu, Kyoto, Japan) simultaneously in positive (detector voltage 1.6 KV) ionization under scan mode. The scan speed of the mass analyzer was 2000 m/z per sec within the range of 400–1000 m/z. A positive full scan mode for screening and library assisted identification was used whereas time schedule selected-ion mode (SIM) in +ve ionization mode of the characteristic abundant adduct ions. Purity of UA was assessed by HPLC and was ≥95% [12]. Chemical shifts are in ppm with reference (internal) to tetramethylsilane (TMS) and J values are in hertz. With the Dept pulse sequence, different types of carbons (C, CH, CH_2_ & CH_3_) in UA were determined. The vacuum liquid chromatographic separations (VLC) were carried out on TLC grade Silica gel H (average particle size 10 µm) purchased from Merck, (Mumbai, India). All the required solvents and reagents were purchased from Spectrochem (Mumbai, India) and Thomas Baker Pvt. Ltd., India. Pre-coated Silica gel (60F) TLC plates 2.5 mm (Merck) were used to determine the profiles of VLC fractions and their purity. The developed TLC plates were first observed at 254 nm in UV and then sprayed with Bacopa reagent [vanillin-ethanol sulphuric acid (1 g: 95 ml: 5 ml)] and spots were visualized after heating the TLC plate at 110°C for 5 minutes.

### Plant material

The leaves of *E. tereticornis* were collected from the medicinal farm of Central Institute of Medicinal and Aromatic Plants (CIMAP), Lucknow, Uttar Pradesh, India during the month of January, 2008. A voucher specimen # 12470 was deposited in the Herbarium section of the Botany and Pharmacognosy Department of the institute.

**Figure 1:**
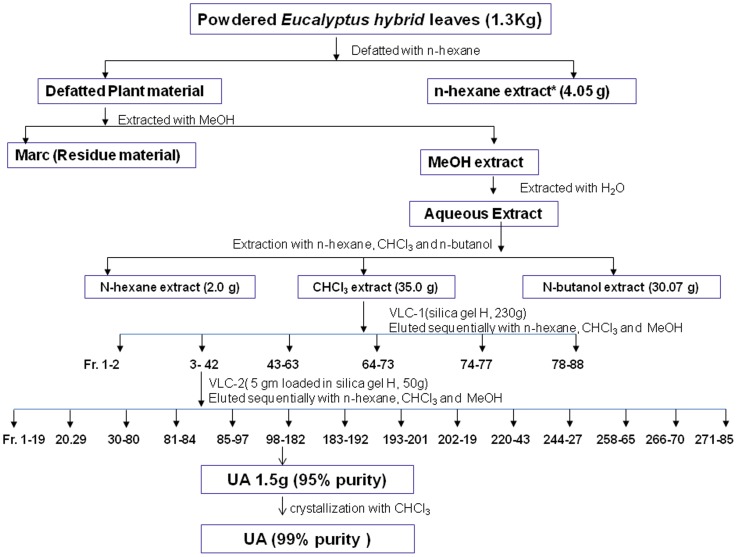
The schematic extraction and fractionation of UA from the leaves of *E. tereticornis*. ^$^Washed with water and the solvent was dried over anhydrous Na_2_SO_4_. ^*^Solvent was completely removed under vacuum at 35°C on a Buchi Rota vapour.

The air dried leaves of *E. tereticornis* (1.3 kg) were powdered and defatted with n-hexane. The defatted leaves were further extracted with MeOH (4×5 L) (Figure 1).The combined MeOH extract was dried under vacuum at 40°C. The MeOH extract so obtained was dissolved in distilled water (2L) and successively fractionated with *n-*hexane, CHCl_3_ and *n-*BuOH (saturated with H_2_O) [12], [13]. All the fractions were evaluated for anti-filarial activity, of which CHCl_3_ fraction (35.0 g) was found active hence subjected for chromatographic separation over VLC-1 using silica gel H (260 g). The gradient elution of VLC was carried out with mixture of hexane, CHCl_3_ and MeOH in increasing order of polarity.

**Figure 2:**
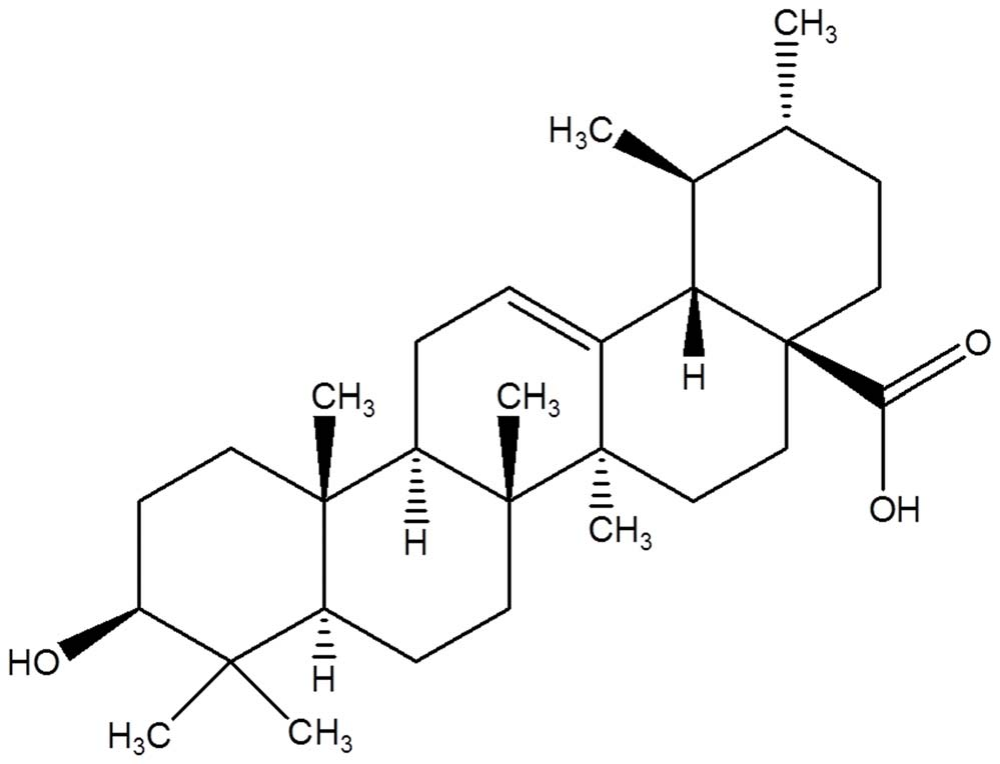
2D structure of Ursolic Acid (UA).

Fractions 3–42 (7.2 g) eluted with hexane- CHCl_3_ (1∶1) to CHCl_3_ – MeOH (99∶1) was a complex mixture. Hence a part of it (5 g) was further chromatographed over VLC-2, using TLC grade silica gel H (50 g). Gradient elution of VLC-2 was carried out with mixture of hexane, CHCl_3_ and MeOH in increasing order of polarity. Fractions 175–182 (1.5 g) eluted with CHCl_3_ (100%) afforded a white amorphous compound (95% pure) which on further crystallization with CHCl_3_ yielded UA (99% pure.). The ^1^H and ^13^C NMR and ESI-MS spectra of the homogenous compound (UA) were recorded and the spectroscopic data are presented as below:

ESI-MS m/z 457 [M+H]^+^, C_30_H_48_O_3_, ^1^H NMR (300 MHz, Pyridine): δ 0.77, 0.78, 0.98, 1.09, 1.14 (3H each, all s, 5 x tert. Me) 0.92 & 0.96 (3H each, each d, J = 6.4 and 7.3 Hz, 2 x sec Me), 2.82 (1H, d, J = 9.9 Hz, H-18 β), 3.20 (1H, dd, J = 6.8 & 8.7 Hz, H-3α). ^13^C NMR (75.5 MHz, Pyridine): C1- 39.5 (t), C2- 28.3 (t), C3- 78.7(d), C4- 39.9 (q), C5- 56.3 (d), C6- 19.1 (t), C 7- 33.9 (t), C8- 40.4 (q), C9- 47.0 (d), C10- 37.7 (q), C11- 23.9 (t), C12- 126.0 (d), C13- 139.6 (q), C14- 42.9 (q), C15- 28.9 (t), C16- 25.2 (t), C17- 48.5 (q), C18- 54.0 (d), C19- 30.5 (d), C20- 39.7 (d), C21- 31.3 (t), C22- 37.6 (t), C23- 29.0 (s), C24-15.8 (s), C25- 16.4 (s), C26- 17.5 (s), C27- 24.1 (s), C28- 179.7 (q), C29- 17.7 (s), C30- 21.4 (s) (Figure 2).

### *In vitro* evaluation of UA/drugs against filarial parasites

Animals: The study was approved by the Institute's Animal Ethics Committee (IAEC) [approval no. 86/09/Para/IAEC; 27/4/09] of CSIR-Central Drug Research Institute, Lucknow, India, under the provisions of CPCSEA (Committee for the Purpose of Control and Supervision on Experiments on Animals), Government of India. All the experiments in animals were conducted in compliance with the IAEC guidelines for use and handling of animals. Throughout the study, jird and *M. coucha* were kept in climate (23±2°C; RH: 60%) and photoperiod (12hr light-dark cycles) controlled animal room. They were fed standard rodent chow supplemented with dried shrimps (*M. coucha*) and had free access to drinking water.

*B. malayi* infection in animals: The human sub-periodic strain of *B. malayi* was cyclically maintained in *M. coucha* [14] and jirds (*Meriones unguiculatus*) [15] through black-eyed susceptible strain of *Aedes aegypti* mosquitoes. Infective larvae of *B. malayi* isolated from experimentally infected *A. aegypti* mosquitoes which were fed on microfilaraemic *M. coucha* (150–200 mf/10 µl blood), were washed thoroughly with insect saline (0.6%). Each animal was inoculated with 100 (*M. coucha*) or 200 L3 (jirds), through subcutaneous (s.c.) and intraperitonial (i.p.) routes, respectively.

Isolation of parasites: Mf and adult worms (female parasites) isolated freshly from peritoneal cavity (p.c.) of jirds harboring 5–6 month old *B. malayi* infection were washed thoroughly in medium Hanks Balanced Salt Solution (HBSS; pH 7.2) containing mixture of antibiotics (penicillin: 100 U/mL; streptomycin: 100 µg/mL) and used for the present study.

### *In vitro* anti-filarial efficacy evaluation

#### Primary evaluation

*In vitro* assays: Based on viability of the parasites, two *in vitro* motility and 3-(4, 5-dimethylthiazol-2-yl)-2,5 diphenyltetrazolium bromide (MTT) reduction assays [16] were carried out for UA. IVM and Diethylcarbamazine-citrate (DEC-C) were used as reference drugs. Incubation medium used was HBSS; pH 7.2 containing mixture of antibiotics as above. For incubation of mf and adult worms cell culture plate (Nunc, Denmark) were used.

The UA and IVM were dissolved in DMSO whereas DEC-C was prepared in sterile triple distilled water (STDW). The antifilarial agents were used at 2-fold serial dilutions ranged from 15.63–1000 µM (DEC), 1.56–100 µM (UA) and 0.31–20 µM (IVM). The final conc. of DMSO in the incubation medium was kept below 0.1%. DMSO (<0.1%) was used in place of test agents solution for control.

Motility assay: Efficacy of the UA and reference drugs was assessed *in vitro* on mf and adult worms of *B. malayi* (as target parasites) using motility (Mf and adult parasite) and MTT (adult parasite only) reduction assay [16], [17]. Duplicate wells containing 40–50 mf/100 µl/well (of 96 well plates) and 1 female worm/ml/well (of 48-well plate) were used. UA (100 µM) or reference drugs IVM (20 µM), or DEC-C (1000 µM) were added to duplicate wells and incubated. Wells with the test compound and DEC were incubated for 24 hr and those with IVM were incubated for 24 and 48 hr as it has a slow action on the parasites. This is the standard protocol followed in our lab [16], [17], [18]. All incubations were at 37°C in 5% CO_2_ atmosphere. The effect on motility of the parasite stages was examined under microscope and scored. The experiment was repeated twice. In case of mf, only motility assay was used.

Motility assessment: Parasite motility was assessed under a microscope after 24/48 h exposure to test substance and scored as: 0 = dead; 1–4 =  loss of motility (1 = 75%; 2 = 50%; 3 = 25% and 4 =  no loss of motility). Loss of motility is defined as the inability of the worms to regain pretreatment level of motility even after incubating in fresh medium *minus* the test agent at 37°C for 1 h. and was expressed as percentage (%) inhibition of control.

MTT- formazan colorimetric assay for viability of worms: The same female worms used in motility were then gently blotted and transferred to 0.1 ml of 0.5% MTT in 0.01 M phosphate-buffered saline (pH 7.2) and incubated for 1 h at 37°C. The formazan formed was extracted in 1 ml of DMSO for 1 h at 37°C and its absorbance was measured at 510 nm in spectrophotometer (PowerWaveX, USA). The mean absorbance value obtained from 4 treated worms was compared with the controls. The viability of the treated worms was assessed by calculating per cent inhibition in motility and MTT reduction over DMSO control worms [16].

Criteria for assessment of *in vitro* hits: 100% inhibition in motility of female adults or mf and or ≥50% inhibition in MTT reduction ability of female parasites was considered acceptable antifilarial (microfilaricidal/adulticidal) activity and picked up as hits and subjected to further testing *in vivo* [17].

#### Secondary evaluation

Determination of IC_50_: For IC_50_ (the concentration at which the parasite motility was inhibited by 50%) determination of the parasites were incubated with two fold serial dilutions from 1.56–100 (UA), 0.31–40 (IVM) and 15.63–1000 µM (DEC-C) using triplicate wells of cell culture plate. Experiments were run in duplicate and incubations were carried out in replicates for 24/48 hr as above. After incubation, inhibition in motility (mf and female worm) and MTT reduction potential of the parasites were assessed as above. The experiment was repeated twice.

Determination of Cytotoxic concentration 50 (CC_50_): The cytotoxicity assay of the test substances was carried out broadly following the method of Pagé et al. [19] with some modifications [20]. Briefly, VERO Cell line C1008 (African green monkey kidney cells) was plated in 96-well plates (Nunc, Denmark) at 0.1×10^6^ cells/ml (100 µl per well) in DMEM supplemented with 10% heat inactivated FBS. A three-fold serial dilution of the test substances (starting from >20 x LC100 conc. of the test agent) in test medium was added. The plates with a final volume of 100 µl/well were incubated in 5% CO_2_ atmosphere at 37°C. After 72 h incubation 10 µl of 0.025% Resazurin in phosphate buffered saline (PBS; pH 7.2) was dispensed as indicator for viability followed by an additional incubation for 4 h and the plate was then read in a fluorescence reader (Synergy HT plate reader, Biotek, USA) at excitation wavelength of 530 nm and an emission wavelength of 590 nm. The assay was run in replicates in each of two independent experiments.

Data of IC_50_ and CC_50_ transferred to a graphic program (Excel) were calculated as described by Page et al. [19] and Mosmann [20] by linear interpolation between the two concentrations above and below 50% inhibition [21].

Selectivity Index (SI) of the UA was computed by the formula as: 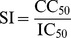



### Molecular modeling and docking studies against glutathione-S-transferase (*Bm*GST) enzyme

**Figure 3:**
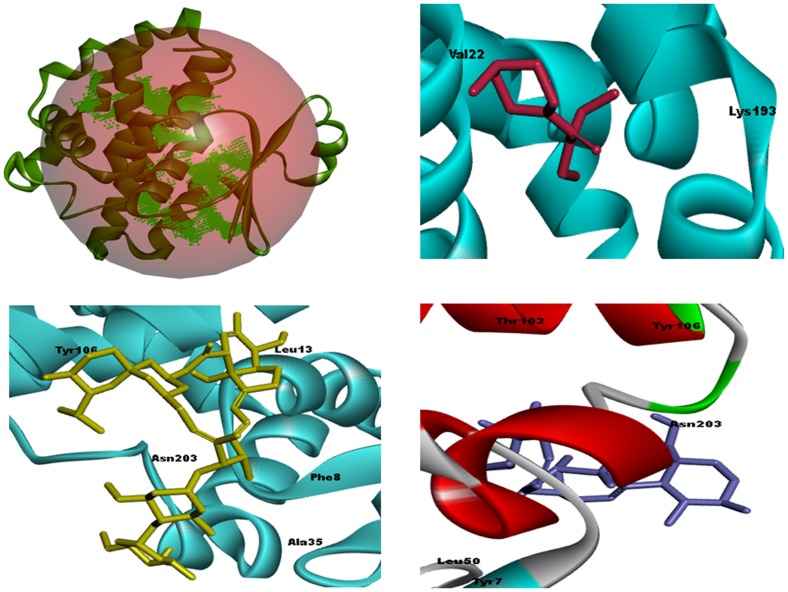
Docking results of studies compounds on *B. malayi* (Filarial nematode worm) glutathione-S-transferase (*Bm*GST) homology model. (a) docked standard drug DEC-c (control) on BmGST model active site with docking energy −4.9 kcal mol^−1^, (b) docked another standard drug Ivermectin (control) with docking energy −8.4 kcal mol^−1^, (c) docked UA on BmGST model with high docking energy −8.6 kcal mol^−1^.

Molecular modeling and geometry cleaning of the UA was performed through ChemBioDraw-Ultra-v12.0 (Cambridge Soft, UK). The 3D structure was subjected to minimized the energy by using molecular mechanics-2 (MM2) force field until the root mean square (RMS) gradient value became smaller than 0.100 kcal mol^−1^ Å. Re-optimization was done by MOPAC (Molecular Orbital Package) method until the RMS gradient attained a value smaller than 0.0001 kcal mol^−1^ Å. The 3D chemical structure of known drugs DEC-c (CID:15432) and IVM (CID: 6321424) were retrieved from PubChem compound database (NCBI, USA). The theoretically solved structure of *B. malayi* glutathione-S-transferase (*Bm*GST) was selected as the potential target for molecular docking simulation studies. The *Bm*GST crystallographic protein 3D structure was retrieved from Protein Data Bank (PDB ID: 1SJO). The Ligsite program was used to identify the potential active site of *Bm*GST model for molecular docking studies and was then cross-checked with template active site as shown in Figure 3 [22]. The visualization studies were executed through Discovery Studio v3.5 (Accelrys Inc., USA, 2013).

### In vivo efficacy

Administration of UA and the reference drugs: The finely powdered UA was suspended in 0.1% Tween-80 prepared in sterilized tap water. Solution of DEC was made in plain STW. *M. coucha* was administered with UA and DEC-c at 100 and 50 mg/kg body weight respectively through i.p. route for 5 consecutive days. The suspensions/solutions of UA/DEC-c were prepared daily before administration to the animals. Control animals received vehicle only.

*B. malayi* -*M. coucha* model: Animals harboring 5–7 months old *B. malayi* infection and showing progressive increase in microfilraemia were used in this study. UA and reference drug treated groups and an equal number of infected untreated animals kept as vehicle treated control, consisted of 5 animals each in two experiments were used.

Mf count in 10 µl blood drawn from tail of the animals between 12:00 noon and 1:00 PM [14] was assessed just before initiation of treatment (day 0), on days 7/8 and 14 post initiation of treatment (p.i.t.) and thereafter at fortnightly intervals till day 84 p.i.t. [17]. The animals were killed on day 91 p.i.t.

Assessment of microfilaricidal efficacy: Microfilaricidal efficacy of UA was evaluated on day 7/8 and 14 p.i.t. and expressed as percent reduction in mf count over pretreatment level [23]–[25].

Assessment of macrofilaricidal and worm sterilization efficacy: Adult worms were recovered from heart, lungs and testes of treated and control animals [14]. Tissues were teased gently and the parasites recovered were then examined under microscope for status of the motility, cell adherence on their surface, dead or calcified worms [23], [24]. Number of worms recovered from the treated and untreated animals was recorded. Macrofilaricidal efficacy of UA and DEC-C was assessed and expressed as percent change in adult worm recovery in treated group over control animals.

All the surviving females were teased individually in a drop of saline to examine condition of intrauterine mf stages of the parasite [23], [24]. Number of sterile female worms recovered from the treated animals was compared with that of control animals and percent sterilization of female worms was determined in treated or control groups over total live female worms recovered from the respective groups.

### Statistical analysis

Statistical analyses were carried out using Statistica version 7/GraphPad Prism 3.0 version software. Results were expressed as mean ± S.D. of data from 5–6 animals in two experiments. The data were subjected to One-way ANOVA analysis and the significance of the difference between means were determined by Newman-Keuls Multiple Comparison Test. P<0.05 was considered significant and marked as *, P<0.01 as highly significant and marked as **, and P<0.001 was very highly significant and marked as ***. The trend analysis was done by fitting the simple regression model (Y = A+BX) using the method of least squares. The slopes of the line were compared by Analysis of Variance.

#### Drug likeness screening studies for ADME/Tox compliance

The ADME/Toxicity parameters compliance was evaluated by screening through Metadrug™, a commercial tool of MetaDiscovery (Thomson Reuters, USA) (http://www.genego.com) [26]. MetaDrug is a system pharmacology or system chemical biology and toxicology platform designed for the assessment of would-be therapeutic indications, off-target effects and potential toxic end points of novel small molecule compounds. In the studied work, this database/tool was used to predict and evaluate the human metabolism compliance, toxicity risk assessment and mode of action by using standard experimental data.

## Results

The leaves of *E*. *tereticornis* were extracted and fractionated, according to the scheme given in Figure 1.

### Worm motility and MTT reduction assay

Of the three extracts tested *in vitro* using worm motility assay, the CHCl_3_ extract killed adult female worms (LC_100_: 400 µM) and mf (LC_100_: 200 µM) (Table 1). The CHCl_3_ extract was subjected to repeated chromatographic separations over VLC using TLC grade silica gel H, which finally resulted in the isolation of a major compound. This major compound on further crystallization with CHCl_3_ afforded 99% pure white crystals. The ^1^H, ^13^C NMR and ESI-MS spectroscopic data of these crystals confirmed that this is a pentacyclic triterpene, ursolic acid (UA) (Figure 1). Finally, UA was tested for its anti-filarial activity against *B. malayi* using *in vitro* assays.

**Table 1:** *In vitro* activity of chloroform extract of *E. tereticornis*, its main constituent Ursolic Acid (UA) and reference drugs ivermectin and DEC on microfilariae and female adult worms of *B. malayi*.

	Effect on female adult worm			Effect on microfilariae (Mf)		CC_50_ c (µM)	SI	
Anti-filarial agent	LC100^a^ (µM) in motility assay (% inhibition)	IC_50_ b (µM) in motility assay	Mean % inhibition in MTT	LC100 (µM) in motility assay (% inhibition)	IC_50_ b (µM) in motility assay		w.r.t. motility of Adults	w.r.t. motility of Mf
CHCl_3_ extract	>100	-	8.55	>100	-	-	-	-
UA	100 (100)	35.36	86.12	25 (90)	8.84	300	8.48	33.94
IVM	5	3.05	5.80	2.5	1.57	250	81.96	159.23
DEC-c*	1000 (100)	314.98	62.54	500 (100)	297.30	8926	28.34	30.02

a LC100 = 100% reduction in motility indicates death of parasite;

b IC_50_ = 50% concentration of the agent at which 50% inhibition in motility is achieved;

c CC_50_ =  concentration at which 50% of cells are killed; SI =  Selectivity Index (CC_50_/IC_50_); w.r.t. =  with respect to; *Diethylcarbamazine citrate.

Further, UA was tested against mf and female adult worms of *B. malayi* using motility and or MTT assays and the results are summarized in Table 1. Like chloroform extract UA was also found to be more effective in killing mf (LC_100_: 50 µM) than adult worms (LC100: 100 µM) and its IC_50_ values were 35.36 and 8.84 µM against the respective parasite stages. UA exerted >86% inhibition in MTT reduction ability of the adult worms. It reduced the viability of female parasite in a gradual dose dependent manner as assessed by MTT reduction assay (**Figure S1**). The CC_50_ (>300 µM) and SI (>10) values of UA demonstrated that it is safe for carrying out *in vivo* screening (Table 1).

The time point studied for the standard drug IVM was 24 hr and 48 hr as IVM has slow action on the parasites. After 48 hr post incubation IVM was effective in inhibiting motility of female adult worm and mf at a minimum conc. of 5.0 µM and 2.5 µM (LC_100_), respectively. Its IC_50_ against adult worms was 3.05 µM and that of mf was 1.49 µM. However IVM was less effective when parasites were incubated for 24 hr. IVM failed to inhibit MTT reduction ability of female worms even after 48 hr incubation (Table 1). On the other hand DEC-C required much higher concentration to kill the female worms (LC_100_: 1000 µM) and mf (LC_100_: 500 µM); it inhibited MTT reduction skill of the adult parasite to the tune of 62.55%. The IC_50_ of DEC against the respective parasite stages were found to be 353.55 µM and 297.30 µM.

Concentration-dependent' LC_100_ and IC_50_ of UA, ivermectin and DEC for microfilariae and adult parasites of *B. malayi* in motility and MTT assays are shown in Figures S1–S3. After 24 h incubation UA (**Figure S1**) and DEC-C (**Figure S2**) caused concentration dependent decrease in viability of the parasites. However, in case of IVM the viability was time and conc. dependent (**Figure S3**).

In summary, *in vitro* findings revealed that CHCl_3_ extract of *E. tereticornis* was microfilaricidal and macrofilaricidal, active against human filarial worm *B. malayi* and the active principal was localized to UA.

### Molecular docking of UA on *Bm*GST

The current status of filaria have paved the way for investigating new lead compounds, which could be useful for the development of anti-filarial agents as there is a persistent urge for a lead to become candidate drug. The enzyme glutathione-s-transferase (GST) is playing a significant role in the long-term existence of filarial worms in mammalian host. The GST enzyme is a well known potential molecular target to inhibit filarial parasite's growth [27], [28]. Therefore, the *Bm*GST theoretical protein structure 3D model was retrieved from PDB crystallographic database and later used for molecular docking simulation studies of UA, to explore the possible mechanism of action within the filarial worm (Table 2). The docking results showed high binding affinity (i.e., low docking energy; −8.6 kcal mol^−1^) similar to that of reference drugs, DEC-c (−4.9 kcal mol^−1^) and IVM (−8.4 kcal mol^−1^). Docking results of UA also showed formation of H-bond (length 3.0 Å) with aromatic hydrophobic residue TYR-106, this may be the reason of high binding affinity, stability and activity of UA. The other binding site amino acid residues within a selection radius of 4 Å from the bound UA against *Bm*GST protein structure model were nucleophilic (polar, hydrophobic) e.g., threonine (THR-102), aromatic (hydrophobic) e.g., phenylalanine (PHE-8), tyrosine (TYR-7, TYR-106), polar amide e.g., asparagine (ASN-203), glutamine (GLN-49), hydrophobic e.g., leucine (LEU-13, LEU-50) (Figure 3). These results suggest that UA interacted well with the conserved hydrophobic amino acid residues of *BmGST*. The molecular docking results showed that UA had significant similarity with respect to interacting amino acid residues and hydrogen bonds to that of the reference drug IVM, while the second reference drug Dec-c showed almost different interacting amino acid residues and hydrogen bond pattern. On the basis of docking binding affinity studies, it may be suggested that UA can be used as a potential lead against lymphatic filarial parasites by targeting GST.

**Table 2:** Details of Docking energy, active site pocket residues and H-bonds revealed by molecular docking of DEC, IVM and UA on *Bm*GST of *B. malayi*.

S. No.	Receptor	Anti-filarial agent	Binding Affinity (kcal/mol)	Interacting Residues	No of H-bonds
1	1SJO	DEC *	-4.9	VAL-22, ILE-26, LYS-189, GLU-190, LYS-193, ARG-195	2.9 = LYS-193
2	1SJO	IVM*	-8.4	PHE-8, LEU-13, ASN-34, ALA-35, LEU-50, TYR-106, ASN-203, ASN-205	2.7 = ASN-203 3.1 = TYR-106
3	1SJO	UA	-8.6	TYR-7, PHE-8, LEU-13, GLN-49, LEU-50, THR-102, TYR-106, ASN-203	3.0 = TYR-106

### ADME/Tox parameters evaluation

Since, docking results showed that UA may act as a potential anti-filarial lead, therefore *in silico* ADME/Tox parameters screening study was performed through Discovery Studio v3.5 molecular modeling & drug discovery software (Accelrys, USA). The UA, DEC and IVM were evaluated with standard descriptors and all the chemical descriptors and parameters of ADME were calculated (Table 3). The ADME results showed that there was no predictive hepatotoxicity and UA was comparable to standard range.

**Table 3:** Predicted ADME parameters (DS v3.5, Accelrys, USA).

Anti-filarial agent	Aqueous solubility	Blood brain barrier penetration	CYP2D6 binding	Hepatotoxicity	Intestinal absorption	Plasma protein binding
DEC	4	2(Medium)	False (non-inhibitor)	True (toxic)	0 (Good)	False (Poorly bounded)
IVM	3	4 (Undefined)	False (non-inhibitor)	True (toxic)	3 (very poor)	False (Poorly bounded)
UA	1	0 (very high penetrant)	False (non-inhibitor)	False (non-toxic)	1 (moderate)	True (Highly bounded)

**Figure 4:**
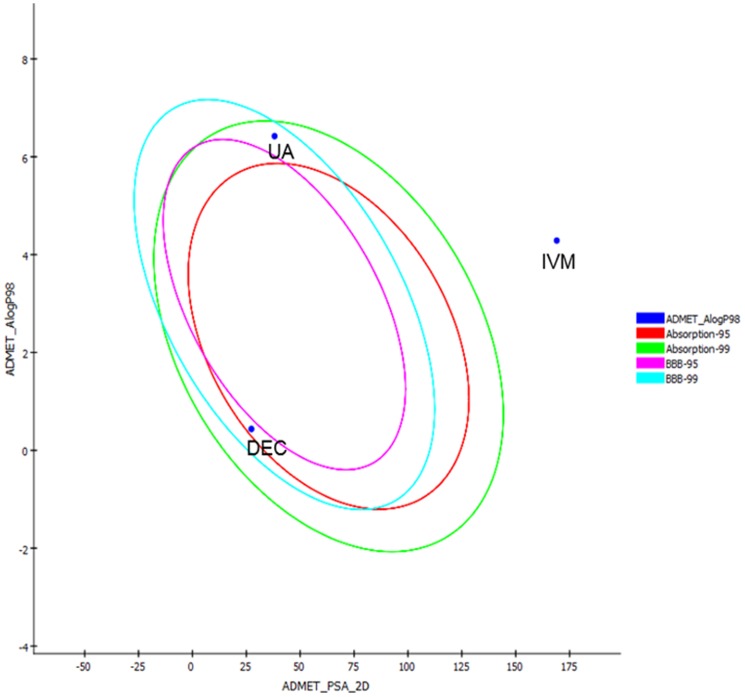
Adsorption model of Ursolic Acid (UA) and the standard antifilarial drugs.

The ADME 2-D graph was plotted against Alogp98 versus PSA_2D (polar surface area) (Figure 4), which showed that the UA and Dec-c were inside the confidence limit ellipses of 99% for the blood brain barrier penetration and human intestinal absorption models compliance. On the other hand, IVM fallen outside the ellipse (undefined) showing very poor absorption and blood brain barrier penetration. Although, UA showed less water solubility, moderate intestinal absorption, but exhibited high plasma protein binding.

Although, ADME results showed that UA violates Lipinski's rule of five due to high logP value (logP>5), hence may cause problem in absorption through biological membranes or intestinal absorption, but it still falls within the acceptable limit of rule of five, when compared with the reference drugs, DEC and IVM (Table 4).

**Table 4:** Compliance of Dec, IVM & UA to the theoretical parameters of oral bioavailability and drug likeness properties.

Anti-filarial agent	Pharmacokinetic properties (ADME) dependent on chemical descriptors								
	ADM	AE	ADME	AD					Lipinski's rule of 5 violation
	Oral bioavailability: TPSA (Å^2^)	MW	logP	H-bond donor			H-bond acceptor		
				NH_2_ group count	-N- group count	OH group count	N atom count	O atom count	
DEC	26.785	199	0.881	0	0	0	3	1	0
IVM	170.095	861	4.076	0	0	3	0	14	2
UA	57.527	456	6.789	0	0	1	0	3	1

Note: A =  absorption, D =  distribution, M =  metabolism, and E =  excretion; TPSA =  topological polar surface area; MW =  molecular weight; Log P =  octanol/water partition coefficient.

### Toxicity risk assessment

The toxicity risk assessment at high doses and/or long term use was evaluated through the OSIRIS web server for the reference drugs, DEC, IVM and the studied compound UA (Table 5). In this screening four important toxicity risk parameters *viz*., mutagenicity, tumorogenicity, skin irritation and reproductive/developmental toxicity parameters were evaluated for high doses or long term use toxicity. The toxicity screening results showed that UA and the reference drug IVM showed no features of risk of tumorogenicity, mutagenicity, reproductive toxicity and skin irritation, therefore UA is safe for human use, whereas DEC yielded a high risk of mutagenicity and reproductive toxicity.

**Table 5:** Details of computational toxicity risk parameters of DEC, IVM and UA calculated by OSIRIS.

	Toxicity risk parameters			
Compound	MUT	TUMO	IRRI	REP
DEC	High Risk	No risk	No risk	High Risk
IVM	No risk	No risk	No risk	No risk
UA	No risk	No risk	No risk	No risk

Note: MUT =  Mutagenicity, TUMO =  Tumorogenicity, IRRI =  Irritation, REP =  Reproduction.

### *In vivo* anti-filarial efficacy

#### *Brugia malayi - M. coucha* model

**Figure 5:**
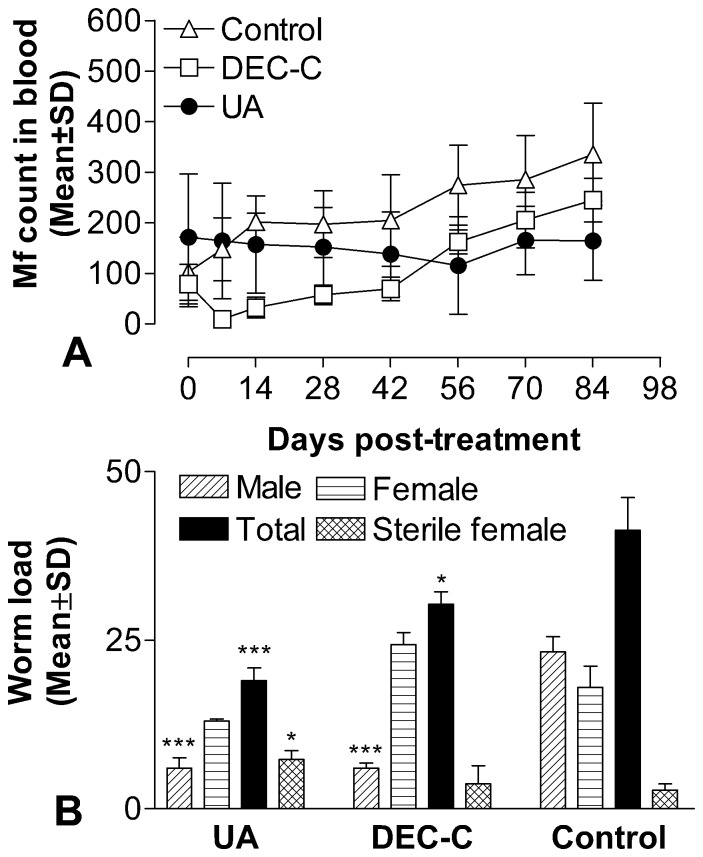
Micro-(A) and macrofilaricidal (B) activity of UA and reference drug diethylcarbamazine-citrate (DEC-C) against *Brugia malayi* in *Mastomys coucha*. Values are mean ± S.D. of 5 animals from two experiments. (A) No alteration in Microfilarial count in treated animals at each time point post initiation of treatment over day 0. Statistics: Student's *‘t’* test. Significance level (B) *P<0.05 (vs sterilized female worm of control animals).

Microfilaricidal activity: Figure 5A shows anti-filarial efficacy of UA against *B. malayi* in *M. coucha* at 100 mg/kg s.c. for 5 consecutive days. UA produced 4–33% lower microfilaremia (statistically not significant) than 0 day throughout the post treatment observation period (Figure 5A). In other words, the microfilaremia in UA treated animals remained below (4–33%) the pretreatment (0 day) level throughout the observation period, while in the untreated control it was (progressively) higher than the pretreatment level and never equaled the 0 day level. This clearly shows that UA possesses considerable antifilarial efficacy. DEC-C (50 mg/kg, s.c. x 5 days), which is principally a microfilaricide, caused >85% reduction in microfilarial count on day 7 p.i.t. which progressively increased and relapsed on day 49 p.i.t.; the count further increased rapidly and crossed the pretreatment level by day 56 p.i.t. The trend of microfilaremia from day 7 to day 84 p.i.t. in the three groups (control untreated, UA treated and DEC treated) against time (post treatment) was determined and compared among each other using linear trend analysis. The baseline of each group was subjected to equality and the observations were converted to show percent change at each time point. The baseline adjusted data was fitted to straight line. The analysis showed that while the mf count in the DEC-treated animals after an initial dramatic drop on day 7 p.i.t., increased gradually over time, it remained almost unchanged with time in UA treated group (trend not significant). Thus UA was found to be better than DEC in controlling microfilaremia. The details are given in File S1: ‘*In vivo* antifilarial efficacy in *Brugia malayi -M. coucha* model: Microfilaricidal activity’.

Macrofilaricidal and embryostatic activity: UA (100 mg/kg, s.c. for 5 days) caused around 54% (P<0.001) adulticidal action over the untreated control. A moderate embryostatic effect of UA (56.15%; P<0.05) was also noticed in female worms (Figure 5B). DEC-C treatment (50 mg/kg, s.c. x 5 days) resulted in 26.47% reduction (P<0.05) in adult worms but did not exert any significant embryostatic effect on female worms when compared to that of untreated control animals (Figure 5B). The general behavior of the treated animals was found normal during entire observation period indicating that UA is safe.

Together, the results of UA showed promising antifilarial activity *in vitro* and *in vivo* with no adverse affect on health and general behavior of the treated animals.

### ADME/Tox compliance

The compound UA was evaluated through MetaDrug tool (Thomson Reuters, USA) for compliance to the standard ADME/Tox parameters. Results showed the information of metabolites, QSAR based prediction of ADME/Tox properties, therapeutic activities, information of analogues, pathways, potential targets and signaling pathway map by leveraging an extensive database of chemical structures and pharmacological activities and visualized in the context of pathways, cell processes, toxicity and disease networks that are perturbed by the compound and its metabolites. These results for UA are briefly discussed below:

Prediction of therapeutic activities for UA: Large numbers of therapeutic activities for the compound UA were identified through MetaDrug tool (Thomson Reuters, USA). The evaluated therapeutic activities for UA were; allergy, Alzheimer, angina, arthritis, asthma, bacterial, cancer, depression, diabetes, HIV, heart failure, hyperlipidaemia, obesity, migraine, osteoporosis and many more. The predicted activities for UA were classified as active or non-active based on calculated values. The predicted properties of UA were calculated on the basis of Tanimoto Percentage [TP] values (standard cut-off ≥0.5) (Table 6).

**Table 6:** Predicted therapeutic activity of UA against various reported diseases.

Property	Model description	Value/(TP)
**Allergy**	Potential antiallergic activity. Cutoff is 0.5. Values higher than 0.5 indicate potentially active compounds. Training set consists of approved drugs. Model description: Training set N = 258	0.50 (60.67)
**Arthritis**	Potential activity against arthritis. Cutoff is 0.5. Values higher than 0.5 indicate potentially active compounds. Training set consists of approved drugs	0.72 (58.72)
**Cancer**	Potential activity against cancer. Cutoff is 0.5. Values higher than 0.5 indicate potentially active compounds. Training set consists of approved drugs. Model description: Training set N = 886	0.69 (64.84)
**Hyperlipidemia**	Potential antihyperlipidemic activity. Cutoff is 0.5. Values higher than 0.5 indicate potentially active compounds. Training set consists of approved drugs. Model description: Training set N = 185	0.91 (66.67)
**Inflammation**	Potential anti-inflammatory activity. Cutoff is 0.5. Values higher than 0.5 indicate potentially active compounds. Training set consists of approved drugs. Model description: Training set N = 598	0.50 (79.31)
**Migraine**	Potential activity against migraine. Cutoff is 0.5. Values higher than 0.5 indicate potentially active compounds. Training set consists of approved drugs	0.62 (97.37)
**Obesity**	Potential activity against obesty. Cutoff is 0.5. Values higher than 0.5 indicate potentially active compounds. Training set consists of approved drugs	0.94 (97.37)
**Osteoporosis**	Potential anti-osteoporosis activity. Cutoff is 0.5. Values higher than 0.5 indicate potentially active compounds. Training set consists of approved drugs	0.80 (97.37)
**Skin Diseases**	Potential activity against skin diseases. Cutoff is 0.5. Values higher than 0.5 indicate potentially active compounds. Training set consists of approved drugs. Model description: Training set N = 255	0.94 (53.04)

Prediction of analogues, pathways and potential targets for UA: The chemical structures and the name of some known similar compounds or analogues were predicted by MetaDrug tool related to UA on the basis of structural similarity (in the range of 98–100%). MetaDrug tool also detected the potential biological pathways and the targets with experimentally known prior mode of action for UA (Table 7).

**Table 7:** The reported interaction between UA and target.

S. No.	Target	Type	Drug	Interactions	Similarity	Effect	Pubmed/Patent ID
1	COX-2 (PTGS2)	§	Ursolic acid	**€**	100	Inhibition	12444669
2	OATP-C	**¥**	Ursolic acid	**£**	100	Inhibition	12871156
3	DNA ligase I	§	(1S, 2R, 4aS, 6aS, 6bR, 10S, 12aR)-10-Hydroxy-1, 2, 6a, 6b, 9, 9, 12a-heptamethyl-1, 3, 4, 5, 6, 6a, 6b, 7, 8, 8a, 9, 10, 11, 12, 12a, 12b, 13, 14b-octadecahydro-2H-picene-4a-carboxylic acid	**€**	100	Unspecified	15519169
4	DNA polymerase beta	§	10-Hydroxy-1, 2, 6a, 6b, 9, 9, 12a-heptamethyl-1, 3, 4, 5, 6, 6a, 6b, 7, 8, 8a, 9, 10, 11, 12, 12a, 12b, 13, 14b-octadecahydro-2H-picene-4a-carboxylic acid (1)	**€**	100	Inhibition	15974441
5	ACAT2	§	(1S, 2R, 4aS, 6aS, 6bR, 8aR, 10S, 12aR, 12bR, 14bS)-10-Hydroxy-1, 2, 6a, 6b, 9, 9, 12a-heptamethyl-1, 3, 4, 5, 6, 6a, 6b, 7, 8, 8a, 9, 10, 11, 12, 12a, 12b, 13, 14b-octadecahydro-2H-picene-4a-carboxylic acid	**€**	100	Inhibition	16462051
6	SOAT1	§	(1S, 2R, 4aS, 6aS, 6bR, 10S, 12aR)-10-Hydroxy-1, 2, 6a, 6b, 9, 9, 12a-heptamethyl-1, 3, 4, 5, 6, 6a, 6b, 7, 8, 8a, 9, 10, 11, 12, 12a, 12b, 13, 14b-octadecahydro-2H-picene-4a-carboxylic acid	**€**	100	Inhibition	16462051
7	SOAT2	§	(1S, 2R, 4aS, 6aS, 6bR, 10S, 12aR)-10-Hydroxy-1, 2, 6a, 6b, 9, 9, 12a-heptamethyl-1, 3, 4, 5, 6, 6a, 6b, 7, 8, 8a, 9, 10, 11, 12, 12a, 12b, 13, 14b-octadecahydro-2H-picene-4a-carboxylic acid	**€**	100	Inhibition	15974441
8	ACAT1	§	(1S, 2R, 4aS, 6aS, 6bR, 8aR, 10S, 12aR, 12bR, 14bS)-10-Hydroxy-1, 2, 6a, 6b, 9, 9, 12a-heptamethyl-1, 3, 4, 5, 6, 6a, 6b, 7, 8, 8a, 9, 10, 11, 12, 12a, 12b, 13, 14b-octadecahydro-2H-picene-4a-carboxylic acid	**€**	100	Inhibition	11794520

§ =  Generic enzymes; **€** =  Unspecified; **£** =  Inhibition is done with unspecified mechanism; **¥** =  Transporter.

**Figure 6:**
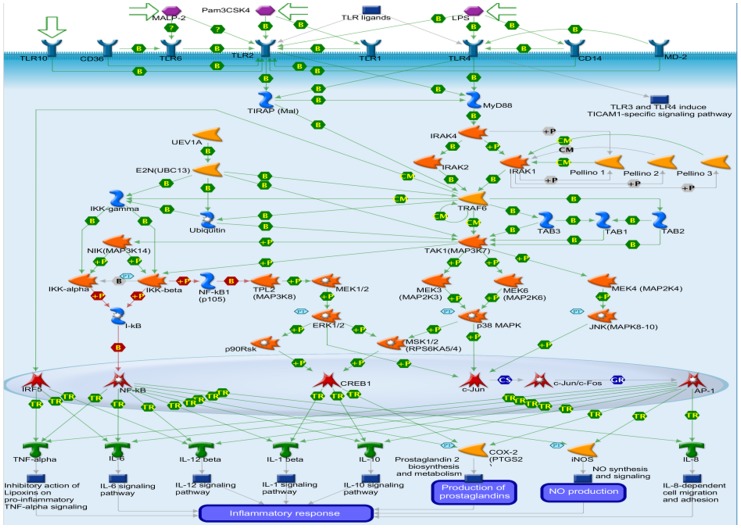
Signaling pathway map screened by Metadrug.

Prediction of metabolic signaling pathway map for UA: Immune response through TLR2 and TLR4 signaling pathways identified through MetaDrug tool on the basis of -lopP value *i.e.*, 1.834e-8 (7.736) with six network objects. TLR2 and TLR4 induce MyD88/IRAK/TRAF6-dependent pathway in target cells, leading to activation of transcription factors NF-kB, AP-1, CREB1 and IRF5, which induce production of various proinflammatory mediators including cytokines, chemokines, nitric oxide (NO) and prostaglandins, leading to inflammatory response (Figure 6).

## Discussion

There are only a few medicinal plant extracts and the isolated molecules, which have shown good anti-filarial activity. The literature showed that some secondary metabolites such as triterpenoids and coumarins showed significant activity against filarial parasites. Our recent finding on the antifilarial activity of pentacyclic triterpenoid, glycyrrhetinic acid (GA) has given us advantage of exploring anti-filarial activity in UA, having similar pentacyclic triterpenoid chemical structure [9], [29]. The UA isolated from the leaves of *E. tereticornis* was in full agreement with the ^1^H, ^13^C NMR and ESI-MS spectroscopic data with the commercially available UA (SIGMA-ALDRICH).

The *in vitro* anti-filarial activity of UA against mf and the adult worms, prompted us to carry out it's *in silico* studies to investigate its possible mechanism of action. It is well known that filarial nematode's detoxify GST enzymes, which play a significant role in the survival of the parasites inside the host's body. This enzyme has effective ability to neutralize the reactive oxygen species (ROS) attack on membrane that acts as cytotoxic products and protect the helminths inside the host [30]–[32]. With this background, the *in silico* molecular docking binding affinity of UA against the GST enzyme was studied. The docking experiments were performed, which showed high binding affinity of UA with *BmG*ST enzyme.

It was observed that for killing the life stages of parasites in vitro, 10 times less concentration of UA was required than the drug DEC. Similarly, in vivo, UA treatment afforded 4–33% drop in microfilaraemia over 0 day throughout the post treatment observation period. While in the untreated control it was (progressively) higher than the pretreatment level and never equaled the 0 day level. The analysis trend in the DEC-treated animals showed that the mf count after an initial dramatic drop, increased gradually over time, while in UA treated animals microfilaraemia remained almost static. This clearly shows that UA was better than DEC in controlling microfilaraemia. Further UA exhibited 54% adulticidal and 56% embryostatic effect with static microfilaraemia while DEC produced ∼26% macrofilaricidal, 15% embryostatic and >85% microfilaricidal effect (on day 7 p.i.t.). These results indicate that UA is clearly superior to DEC with respect to macrofilaricidal and embryostatic effect though not with respect to microfilaricidal effect. It may be mentioned here that macrofilaricidal and embryostatic effect of UA were probably responsible for the low and static microfilaraemia. Thus, UA is better than DEC both *in vitro* and *in vivo* in its antifilarial activity. Further UA being a natural compound, has the possibility of lead optimization by QSAR approach. Thus, in view of potential antifilarial activity, absence of toxicity and favorable pharmacokinetics UA may be considered as a suitable lead for designing and development of a safe and effective antifilarial agent.

## Supporting Information

Figure S1 **LC_100_ and IC_50_ of Ursolic acid (UA) for microfilariae and adult parasites of** ***Brugia malayi*** **.** After incubation with UA for 24 h the viability of parasite was assessed in motility assay using mf (**A**) and adult female worms (**B**) and in MTT reduction assay using adult female worms (**C**).
(TIF)

Figure S2 **LC_100_ and IC_50_ of diethylcarbamazine citrate (DEC-C) for microfilariae and adult parasites of** ***B. malayi*** **.** After incubation with DEC-C for 24 h the viability of parasite was assessed in motility assay using mf (**A**) and adult female worms (**B**) and in MTT reduction assay using adult female worms (**C**).
(TIF)

Figure S3 **LC_100_ and IC_50_ of ivermectin for microfilariae and adult parasites of** ***B. malayi*** **.** After incubation with ivermectin for 24 h (**A–C**) and 48 h (**D–F**) the viability of parasite was assessed in motility assay using mf (**A, D**) and adult female worms (**B, E**) and in MTT reduction assay using adult female worms (**C, F**).
(TIF)

File S1 **Linear Trend analysis of mf count data.**
(DOC)
