# Supplementary figures and images for: In Vitro, In Silico and In Vivo Studies of Ursolic Acid as an Anti-Filarial Agent
Source: PLoS One. 2014 Nov 6;9(11):e111244. doi: 10.1371/journal.pone.0111244 (PMC4222910; doi:10.1371/journal.pone.0111244)

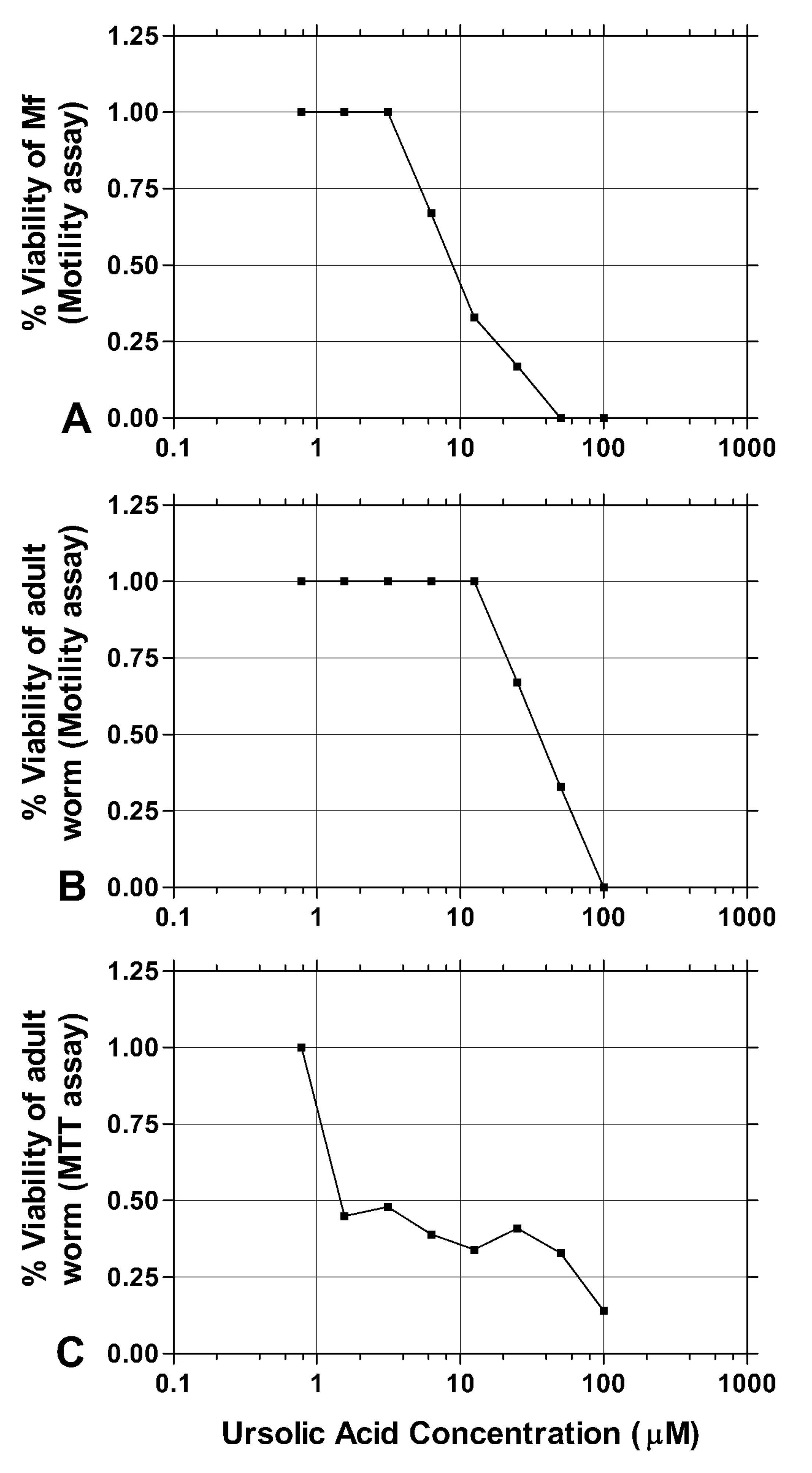

Supplement: Figure S1 — LC100 and IC50 of Ursolic acid (UA) for microfilariae and adult parasites of Brugia malayi . After incubation with UA for 24 h the viability of parasite was assessed in motility assay using mf (A) and adult female worms (B) and in MTT reduction assay using adult female worms (C). (TIF) [file pone.0111244.s001.tif]

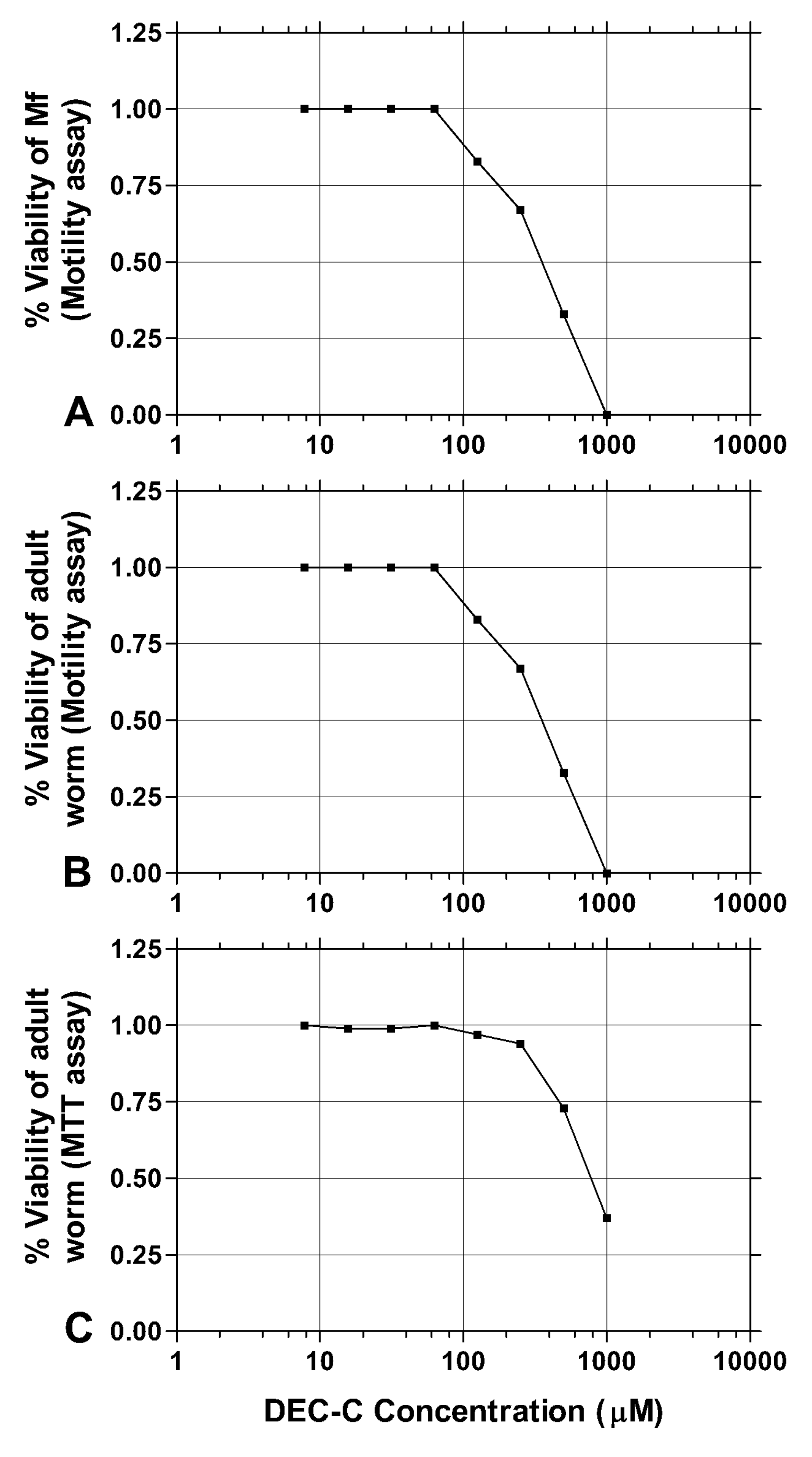

Supplement: Figure S2 — LC100 and IC50 of diethylcarbamazine citrate (DEC-C) for microfilariae and adult parasites of B. malayi . After incubation with DEC-C for 24 h the viability of parasite was assessed in motility assay using mf (A) and adult female worms (B) and in MTT reduction assay using adult female worms (C). (TIF) [file pone.0111244.s002.tif]

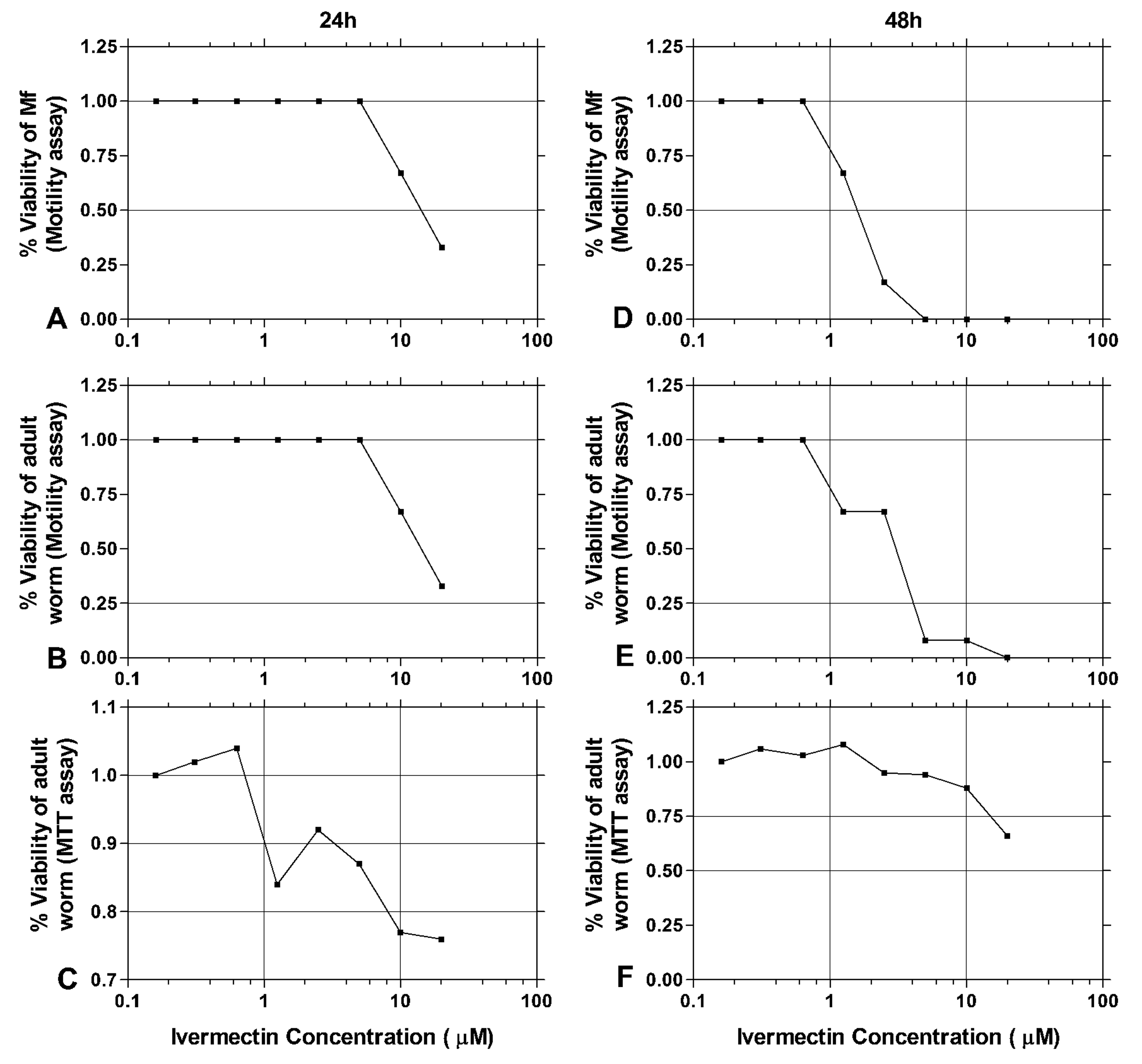

Supplement: Figure S3 — LC100 and IC50 of ivermectin for microfilariae and adult parasites of B. malayi . After incubation with ivermectin for 24 h (A–C) and 48 h (D–F) the viability of parasite was assessed in motility assay using mf (A, D) and adult female worms (B, E) and in MTT reduction assay using adult female worms (C, F). (TIF) [file pone.0111244.s003.tif]
