# Supplementary material for: In Vitro, In Silico and In Vivo Studies of Ursolic Acid as an Anti-Filarial Agent
Source: PLoS One. 2014 Nov 6;9(11):e111244. doi: 10.1371/journal.pone.0111244 (PMC4222910; doi:10.1371/journal.pone.0111244)
Supplement: File S1 — Linear Trend analysis of mf count data. (DOC) [file pone.0111244.s004.doc]

**Supplementary Information**

**Results:**

*In vivo* antifilarial efficacy in *Brugia malayi -M. coucha* model:

Microfilaricidal activity

**Linear Trend analysis of mf count data:** The trend of three groups (control untreated, UA treated and DEC treated) against time (post treatment) was compared among each other. The baseline of each group was subjected to equality. The observations were converted to show percent change at each time point. The baseline adjusted data was fitted to straight line. To determine the effect of the treatment from day 7 to day 84 post initiation of treatment (p.i.t.) (post treatment regime) we used linear trend analysis. Table S1 shows linear fit with intercept and slope of three groups. It shows that the trend of microfilaraemia increased significantly (P<0.001) in the control and DEC treated groups while it was not significant (P>0.05) in the UA treated group.

**Table S1: Linear fit with intercept and slope of three groups of animals**

|  | **Regression Line** | **p-value of Slope** | **Correlation Coefficient (r)** | **Significance** |
| --- | --- | --- | --- | --- |
| Control | Y = -38.48-2.11 Day | 0.0007 | -0.957 | ** |
| UA | Y = 11.10 + 0.21 Day | 0.9044 | 0.056 | NS |
| DEC | Y = 129.95 – 4.00 Day | 0.0001 | -0.980 | ** |

 **= P<0.01; NS: P>0.05

The slopes of the three groups were compared. Analysis of variance showed the three slopes to differ significantly (P<0.001). It means that the rate of change of microfilaraemia in the three groups is different. Individual comparison of slopes between control and UA treated group, & control and DEC treated group showed a significant difference. A significant difference (P<0.001) was also found in the slopes of UA treated and DEC treated groups. A non significant trend in the UA-treated group shows that the mf level was maintained throughout the study period from day 7 p.i.t. onwards. In other words, UA has controlled the mf count from day 7 p.i.t. onwards. On the other hand, DEC treated animals showed a reduction in mf count on day 7 p.i.t. which progressively increased and relapsed (Figure 5) but thereafter, the count increased rapidly. The high value of correlation shows that the mf count increased linearly from day 7 to day 84 p.i.t. in DEC treated and untreated control animals.

This shows that while the mf count in the DEC treated animals increased gradually with every passing day, it remained unchanged with time (trend was not significant) in UA treated group. Thus, UA is better than DEC in controlling microfilaraemia.
